# Supplementary material for: Retrospective Validation of a 168-Gene Expression Signature for Glioma Classification on a Single Molecule Counting Platform
Source: Cancers (Basel). 2021 Jan 25;13(3):439. doi: 10.3390/cancers13030439 (PMC7865579; doi:10.3390/cancers13030439)
Supplement: Supplementary file 1 [file cancers-13-00439-s001.zip › cancers-1055027-suppl-proofreading/cancers-1055027-suppl-proof.docx]

Supplementary Materials: Retrospective Validation of a 168-Gene Expression Signature for Glioma Classification on a Single Molecule Counting Platform

Paul Minh Huy Tran, Lynn Kim Hoang Tran, Khaled bin Satter, Sharad Purohit, John Nechtman, Diane I. Hopkins, Bruno dos Santos, Roni Bollag, Ravindra Kolhe, Suash Sharma and Jin-Xiong She


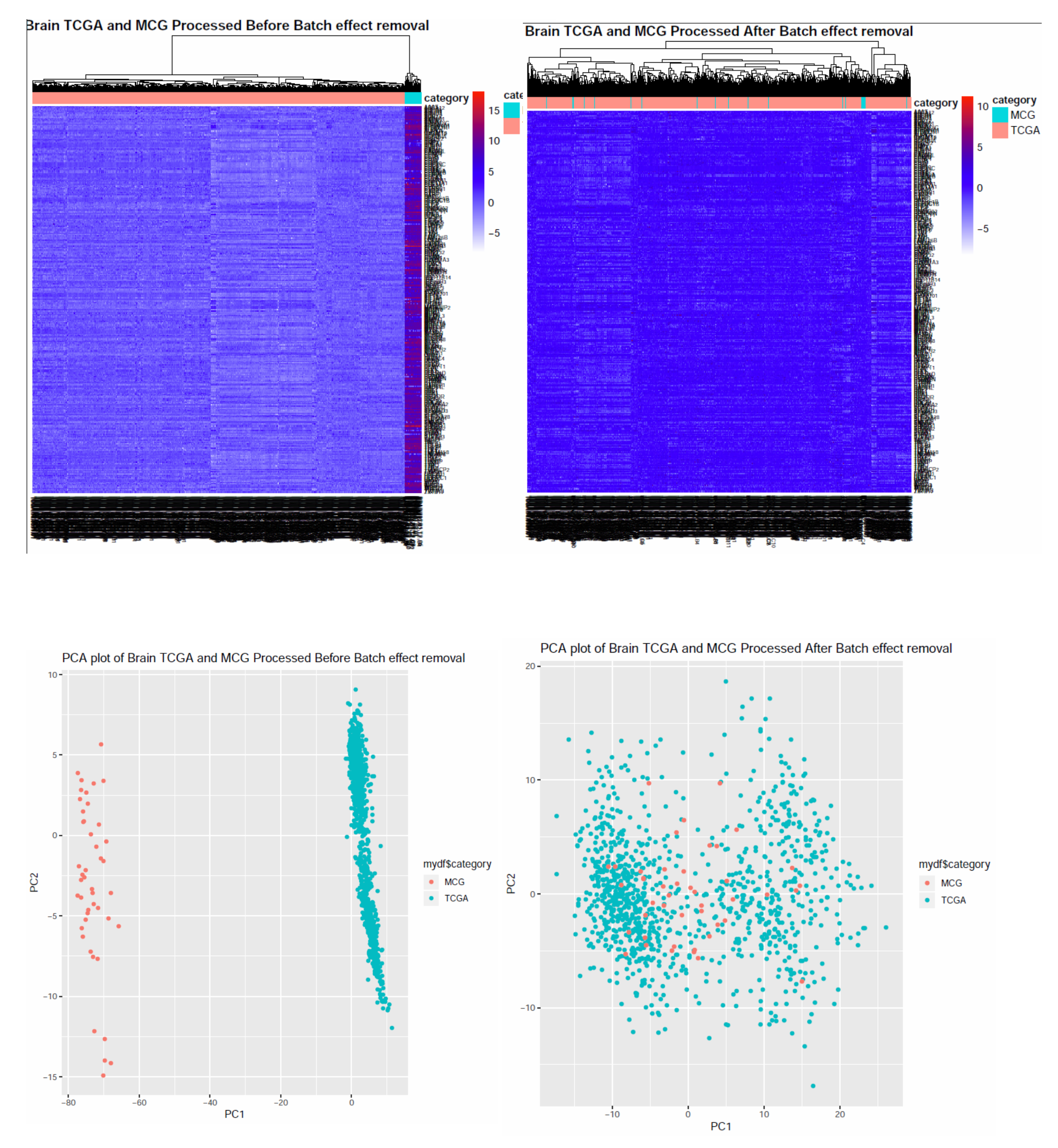


**Figure S1.** PCA plot of TCGA RNASeq and AU Nanostring Data before and after Empirical Bayes Normalization.


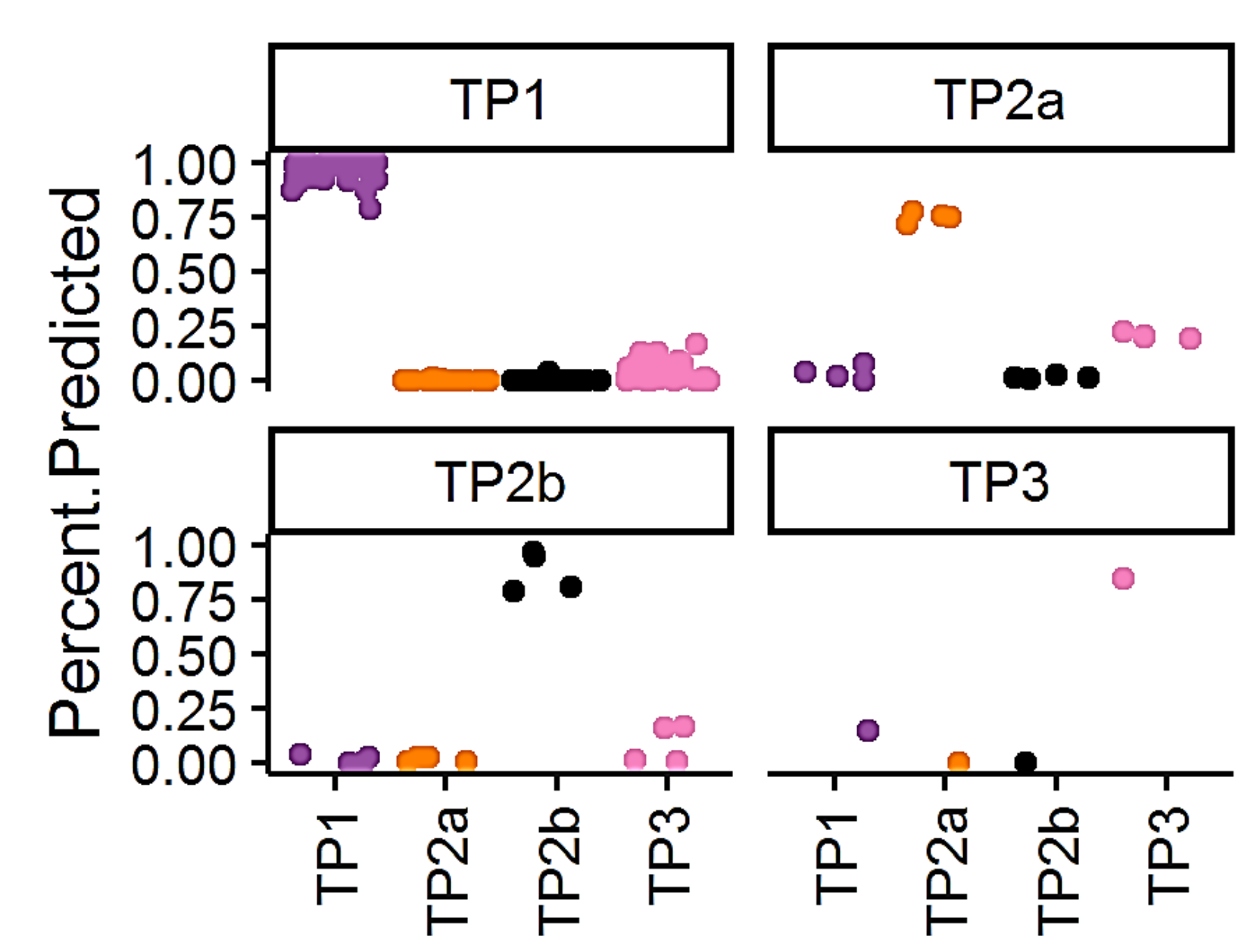


**Figure S2.** Scatter plots showing the percent of the 1000 models which predict the sample as belonging to each classification.
